# NSD Overexpression in the Fat Body Increases Antimicrobial Peptide Production by the Immune Deficiency Pathway in *Drosophila*

**DOI:** 10.3390/ijms24098443

**Published:** 2023-05-08

**Authors:** Chihyun Won, Kyungju Nam, Donghee Ko, Byungjun Kang, Im-Soon Lee

**Affiliations:** Department of Biological Sciences, Konkuk University, Seoul 05029, Republic of Korea

**Keywords:** NSD, SOTOS, IMD, *Drosophila* AMP, NF-κB

## Abstract

Nuclear receptor-binding SET domain-containing protein 1 (NSD1) inactivation in tumor cells contributes to an immune-cold phenotype, indicating its potential association with immune disturbances. *Drosophila* NSD is a homolog of the human NSD1. Thus, in this study, we investigated the effect of NSD overexpression in the fat body, the central organ involved in *Drosophila* immune responses. Upon ectopic expression of NSD in the fat body, the mRNA levels of antimicrobial peptides increased. Using reporter constructs containing deletions of various NF-κB sites in the Attacin-A (*AttA*) promoter, we found that transcriptional activation by NSD is mainly mediated via the IMD pathway by activating Relish. Since the IMD pathway is required to resist Gram-negative bacterial infections, we further examined the effect of fat body-specific NSD overexpression on *Drosophila* immune defenses. Upon oral ingestion of Gram-negative *Pseudomonas entomophila*, the survival rate of the NSD-overexpressing larvae was higher than that of the wild type, suggesting a positive role of NSD in immune responses. Taken together, these results suggest the association of NSD with the IMD pathway and is thus expected to contribute to the elucidation of the molecular mechanisms of immune malfunction in various NSD1-associated human diseases.

## 1. Introduction

Histone methylation is the modification of certain amino acids in histone proteins. Nuclear receptor-binding SET domain protein 1 (NSD1) is a histone methyltransferase that mediates histone H3 methylation at lysine 36 (H3K36) [1]. Although H3K36 methylation was initially reported to be associated with the transcription of active euchromatin, it has also been implicated in diverse roles in many important developmental processes and diseases. In humans, haploinsufficiency of NSD1 causes Sotos syndrome (SOTOS, OMIM #117550), a well-defined childhood overgrowth disease with macrocephaly, advanced bone age, intellectual disability, and characteristic facial features [2,3,4,5]. In contrast, an extra copy of *NSD1* results in the reverse SOTOS phenotype [6,7,8,9].

Notably, patients with SOTOS, mainly caused by NSD1 loss-of-function, frequently exhibit recurrent upper respiratory tract infections [10]. In addition, NSD1 is inactivated in certain types of tumors, resulting in an immune-cold phenotype characterized by a low infiltration of tumor-associated leukocytes [11], suggesting a potential association between NSD1 and immune disturbances.

Recent studies have shown that the innate immune system is epigenetically regulated in mammals and insects [12]. Unlike mammals, which have both innate and adaptive immunity, *Drosophila* flies have only innate immunity [13] and clear infection through a complex inflammatory response involving both cellular and humoral defenses. The fat body is the central organ in *Drosophila* responsible for the humoral response, which synthesizes and secretes numerous antimicrobial peptides (AMPs) into the hemolymph [14,15]. Infection of the fly initiates AMP secretion by one of two distinct signaling cascades, the Toll or immune deficiency (IMD) pathways, depending on the pathogen [16]. The Toll pathway is activated by peptidoglycan from Gram-positive bacteria or fungal glucans and is related to Dorsal and Dorsal-related immunity factors (DIF), whereas the IMD pathway is activated by lipopolysaccharides present in Gram-negative bacteria and induces proteolytic cleavage of another NF-κB-like factor, Relish.

*Drosophila* NSD is a human NSD1 homolog [17,18]. NSD overexpression in the whole body of *Drosophila* causes developmental disruption and prepupal death [19]. In addition, overexpression of NSD in glial cells induces apoptotic cell death and decreases lipid levels in the brain [20]. In this study, we examined the effect of NSD overexpression in the fat body of *Drosophila* on the production of various types of AMPs and performed an oral ingestion experiment with pathogenic Gram-negative bacteria to gain insight into the functions of NSD1 in the immune system of *Drosophila*.

## 2. Results

### 2.1. Generation of a Fat Body-Specific NSD-Overexpressing Fly Using the Gal4/UAS System

**Figure 1:**
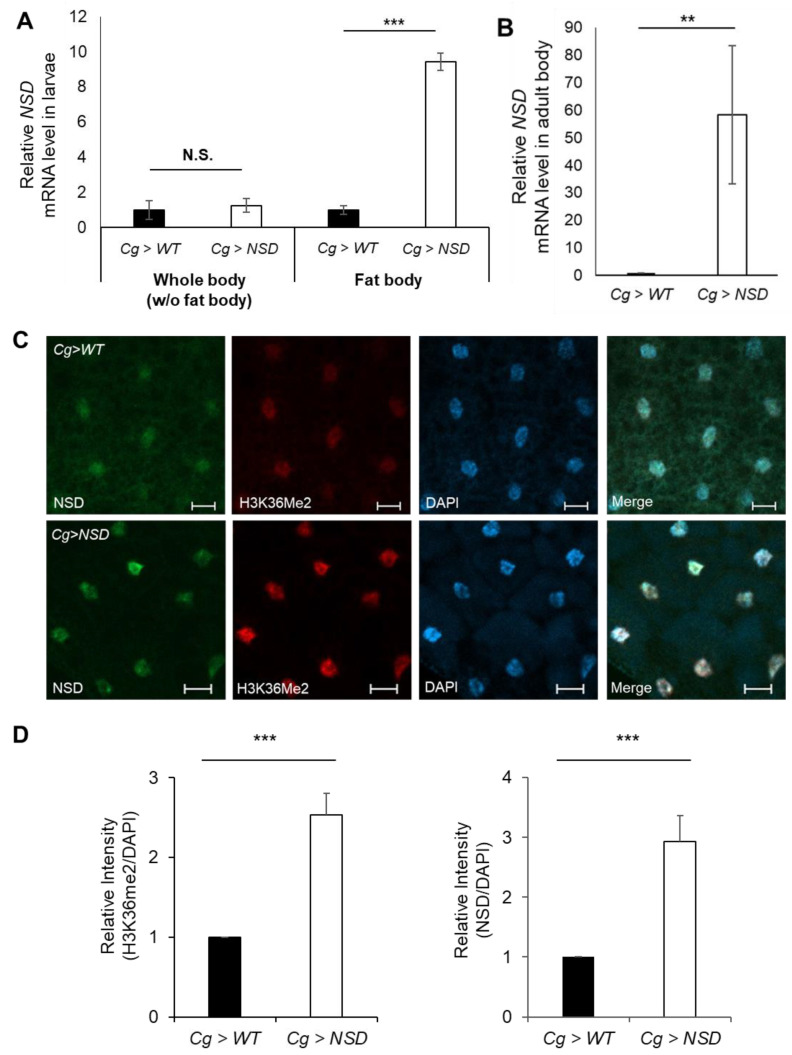
Targeted overexpression of nuclear receptor-binding SET domain protein 1 (NSD) in the *Drosophila* fat body. (**A**) Relative levels of NSD mRNA in the fat body and the fat body-excluded whole body of the third instar larvae expressing *Cg-Gal4*-driven NSD compared to those of the control fly. (**B**) Relative level of NSD mRNA of the NSD-overexpressing whole body from the adult fly. (**C**) Immunofluorescence assay of the NSD-overexpressing third instar larval fat body with anti-NSD and anti-H3K36Me2 antibodies. Scale bar represents 20 μm. (**D**) Quantification of fluorescence intensity data from (**C**). For analysis, nuclear areas are selected, and their mean intensity was measured using ImageJ software (>24 cells over three independent experiments). The intensities were normalized to the DAPI value. Statistical significance was determined via the Mann–Whitney U test (N.S. denotes no-significance, ** *p* < 0.05, *** *p* < 0.005). Graphs represent the mean ± SD.

The fat body of *Drosophila* is the central organ responsible for the immune response against pathogens [21]. Given that the *Cg-Gal4* driver can target expression in the *Drosophila* fat body in both larval and adult males and females [22,23], we generated a fat body-specific NSD-overexpressing fly by mating fat body-specific *Cg-Gal4* flies with *UAS-NSD* flies. We used *Cg-Gal4* flies mated with *w1118* flies as a control for comparison. To determine whether NSD is specifically overexpressed in their fat body, we performed a quantitative polymerase chain reaction (qPCR) on the third instar larval fat body. The results showed a 9.2-fold increase in NSD mRNA levels in the fat body of NSD-overexpressing flies but not in the rest of the larval body (Figure 1A) compared to control flies, confirming specific overexpression of NSD mRNA in this tissue. In addition, the successful overexpression of NSD mRNA in adult flies was confirmed (Figure 1B).

NSD functions as a histone methyltransferase, specifically catalyzing H3K36 dimethylation (H3K36Me2). Thus, to determine whether the NSD protein overproduced in the Cg-NSD fly fat body is biochemically functional, we examined H3K36Me2 and NSD levels using an immunofluorescence assay. Analysis of the third instar larval fat body of the NSD-overexpressing flies showed 2.5-fold and 2.9-fold higher levels of H3K36Me2 and NSD, respectively, compared to the control flies (Figure 1C,D). These results confirm that NSD driven by *Cg-Gal4* is specifically overexpressed in the fat body and that its protein is functional.

### 2.2. NSD Overexpression in the Drosophila Fat Body Affects the Innate Immune System via Relish

#### 2.2.1. Effects of the Fat Body-Targeted NSD Overexpression on the Transcription of Various AMPs

**Figure 2:**
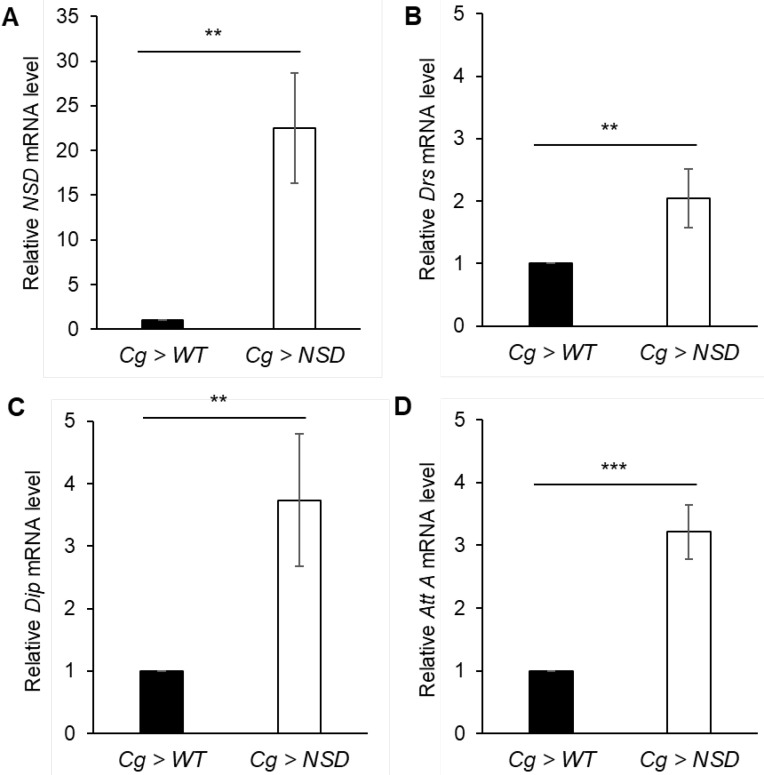
Relative mRNA levels of AMPs from the larval fat body of the NSD-overexpressing fly. (**A**) Relative levels of *NSD* mRNA. (**B**–**D**) Relative levels of Drosomycin (*Drs*), Diptericin (*Dip*), and Attacin-A (*AttA*) mRNAs. Compared to those of the control flies, the fat body of the NSD-overexpressing flies showed increased expression in all three tested AMPs, among which the transactivational level of *Dip* was the highest. All data were normalized with the *Rp49* mRNA. Statistical significance was determined via the Mann–Whitney U test (** *p* < 0.05, *** *p* < 0.005). Graphs represent the mean ± SD.

The immune response in *Drosophila* requires activation of AMP secretion. The Toll and IMD pathways reportedly activate the secretion of different sets of AMPs from the fly fat body upon infection [24,25]. In the transcriptome analysis of ectopic overexpression of NSD in the wing imaginal disc of the fly, we previously observed a disturbance in the transcription of various AMPs (unpublished data). The fat body is a major source of circulating AMPs in the infected flies. Thus, we chose three target genes, the major targets of the two pathways, Drosomycin (*Drs*) of the Toll pathway and Diptericin (*Dip*) of the IMD pathway, respectively, and Attacin-A (*AttA*), which responds to both pathways [26], and investigated the effect of NSD overexpression in the fat body on AMP production. Upon analysis of the mRNA, the fat bodies of the NSD-overexpressing flies showed increased expression of all three tested AMPs. Notably, however, the increase in those associated with the IMD pathway displayed relatively higher transcriptional levels (Figure 2), such that *Dip* and *AttA* mRNAs increased by 3.75- and 3.25-fold, respectively, compared to a 2.1-fold increase in the *Drs* mRNA, indicating more severe deregulation of the IMD pathway due to NSD overexpression.

#### 2.2.2. Activation of the NF-κB Activity by NSD Overexpression

**Figure 3:**
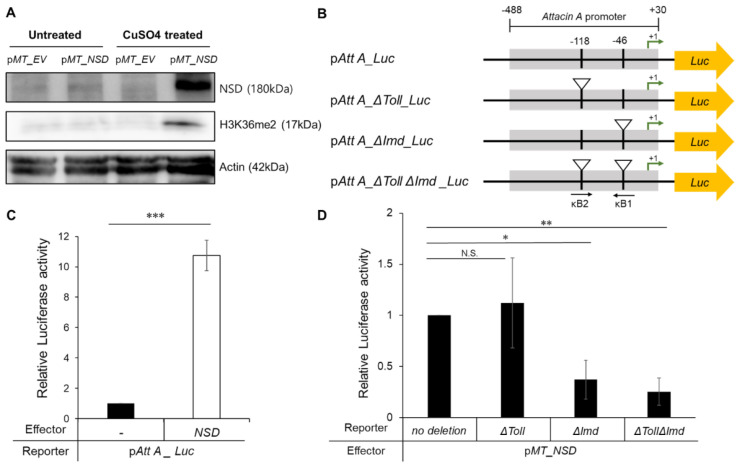
Transient transfection of NSD with the *AttA* promoter containing deletions in NF-κB binding sites into *Drosophila* Schneider 2 (S2) cells. (**A**) Western blot analysis to confirm the induction of NSD expression. To induce its expression from the *MT* promoter-driven vector, cells were treated with copper sulfate to induce NSD expression from the *MT* promoter-driven vector. (**B**) *AttA* promoter-driven reporter constructs used for the experiment. The NF-κB motifs at positions −46 and −118 in the *AttA* promoter are denoted as κB1 (GGGGAAGAAC) and κB2 (GGGGAATTTC), respectively. (**C**) Transcriptional activation of the *AttA* promoter by NSD. (**D**) Downregulation of NSD-mediated transactivation of the *AttA* promoter upon the deletion of κB1 that responds to the immune deficiency (IMD) pathway. Statistical significance was determined via the Mann–Whitney U test (N.S. denotes no-significance, * *p* < 0.5, ** *p* < 0.05, *** *p* < 0.005). Graphs represent the mean ± SD.

Both the Toll and IMD pathways depend on the activation of NF-κB signaling in the *Drosophila*’s innate immunity. As shown in Figure 2, the effect of ectopic NSD expression in the fat body on the mRNA levels displayed a relatively higher preference for IMD. Thus, we further investigated the effect of NSD overexpression on the two innate immune pathways using the *AttA* promoter in the cell-based system because it contains multiple Rel/NF-κB binding sites, among which the κB motifs at positions −46 and −118 have been revealed to be necessary for activation by IMD and Toll, respectively [26]. First, we cloned the *Drosophila* metallothionein promoter (*MT*)-driven NSD expression vector (p*MT-NSD*) for its high and inducible expression, then confirmed by western blot analysis that the NSD protein was efficiently induced by treatment with copper sulfates and was biochemically functional (Figure 3A). Upon co-transfection of the NSD expression vector with the intact *AttA* promoter-driven luciferase, p*AttA*_*Luc*, into Schneider 2 (S2) cells, NSD dramatically transactivated the *AttA* promoter by up to 10-fold in the presence of the inducer, copper sulfate (Figure 3C).

The *AttA* promoter region contains two κB binding sites at positions −46 and −118, which enable a response to the IMD and Toll pathways, respectively [26]. Thus, to determine the κB site of the transcriptional upregulation of *AttA* by NSD, we cloned a set of mutant *AttA* promoter-driven reporters containing deletions in the two κB-motifs, denoted as κB1 and κB2, respectively, using site-directed mutagenesis (Figure 3B). Upon evaluating the function of the motif in directing the expression of the mutant *AttA* reporter gene by transient transfection, we found that the κB1 site at −46 preferentially mediates transactivation by NSD, which responds more strongly to IMD (Figure 3D). In addition, the double deletion at both sites revealed a reduction comparable to κB1 deletion only (Figure 3D), suggesting that transactivation of the *AttA* promoter by NSD is primarily associated with the IMD pathway.

**Figure 4:**
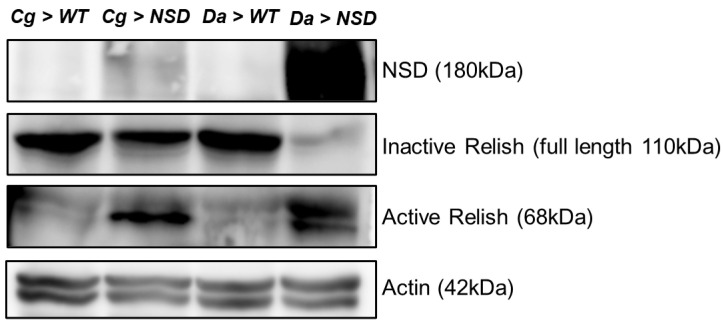
Effect of ectopic expression of NSD on Relish activation. The cell extracts were prepared from the fat body (*Cg* fly lines) and the larval whole body (*Da* fly lines) of wandering third instar larvae to perform western blot analysis with antibodies against the NSD protein, the inactive full-length form of Relish, the cleaved active form of Relish, and actin, respectively.

IMD signaling mainly modulates the activity of NF-κB factor Relish. Full-length Relish (~110 kDa) is composed of an N-terminal Rel homology domain and a C-terminal IκB-like domain and exists in an inactive form in the cytosol under normal conditions. However, in response to Gram-negative bacteria, the inactive full-length Relish is cleaved to generate fragments of approximately 68 and 49 kDa, corresponding to the N- and C-terminal fragments, respectively. As a result of the elimination of the C-terminal IκB region and the N-terminal fragment, the active form of Relish translocates to the nucleus, where it binds to the promoters of AMP genes [27]. Thus, we examined whether Relish is activated by ectopic expression of NSD. The overexpression of NSD in the larval fat body as well as the larval whole body induced a decrease in the levels of full-length Relish in both the targeted tissues (1.6-fold in the fat body; 5.2-fold in the whole body; Figure 4), while the levels of active Relish were increased in the western blot analysis with antibodies specific to the C-terminal and the N-terminal of Relish, respectively. These results suggest the involvement of NSD in the cleavage of Relish for activation.

### 2.3. Enhanced Survival of NSD-Overexpressing Larvae upon Ingestion of Pathogenic Gram-Negative Pseudomonas Entomophila

*P. entomophila* (*Pe*) is a Gram-negative entomopathogenic bacterium that is fatal to *Drosophila* upon oral ingestion [24]. The IMD pathway responds to Gram-negative bacterial infections, whereas the Toll pathway is activated by Gram-positive bacteria and fungi. Because IMD signaling is likely to be upregulated to a greater degree than Toll signaling by NSD overexpression, we next investigated the effect of overexpressing NSD specifically in the fat body of *Drosophila* larvae. To assess the effect of this overexpression on larval survival, we conducted a feeding assay using Gram-negative *Pe* as described in the Materials and Methods section.

**Figure 5:**
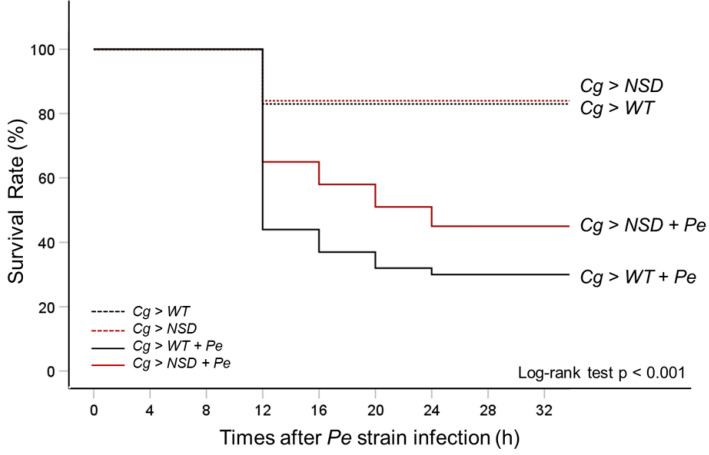
Comparison of survival rates between the NSD-overexpressing *Drosophila* larvae and the control following *Pseudomonas entomophila* (*Pe)* ingestion. We performed a Kaplan–Meier survival analysis of third instar larvae infected with *Pe*. Each group of 100 larvae was infected with *Pe*. Experiments were independently repeated three times, and the pooled data were used to build the survival curves (*n* = 300). Unchallenged negative control groups were treated with fresh LB medium. Statistical significance was measured using the log-rank test, with a *p*-value of < 0.001 (chi-square 110.235), compared to the control. The control and the NSD-overexpressing larvae were naturally infected with *Pe*, depicted as a black solid line (*Cg > WT-Pe*) and a red solid line (*Cg > NSD-Pe*) on the graph, respectively. The relative survival rates of the unchallenged control (*Cg > WT*) and NSD-overexpressing larvae (*Cg > NSD*) are depicted as a black dotted line and a red dotted line, respectively. The quantities of surviving and dead flies were recorded at indicated intervals of 0, 12, 16, 20, 24, and 32 h. After 32 h, the survival rate of larvae was not measured because the remaining larvae had pupated. The *p*-value is for the log-rank test.

We then employed the Kaplan–Meier log-rank approach, which is a widely used statistical method for analyzing survival data, to evaluate whether there were any significant differences in survival between the group of *Drosophila* larvae with fat body-specific NSD overexpression and the control group, particularly under conditions of Gram-negative *Pe* exposure. The results of the oral *Pe* ingestion test showed that NSD-overexpressing *Drosophila* larvae have a significantly better survival rate than the control (the actual log-rank *p*-value < 0.001) (Figure 5); the group with NSD overexpression had a higher survival rate (65%) compared to the control group (44%) at 12 h post-infection with *Pe*. Furthermore, the more resistant pattern of the NSD-overexpressing larvae against *Pe* infection remained throughout the infection experiment, up to 40 h. *Pe* infections killed 70% of the control larvae within 40 h, whereas the NSD larvae died at a slower rate (57% lethality at 40 h). On the other hand, in the absence of *Pe* challenge, the survival rates of the NSD-overexpressing larvae were similar to those of the control larvae. Thus, the higher survival rates in the NSD overexpression group may indicate that NSD plays a role in improving the larvae’s ability to tolerate *Pe* infection by enhancing the immune response, possibly through the IMD pathway, resulting in increased survival and defense against Gram-negative bacteria, compared to the control group.

## 3. Discussion

*Drosophila* flies do not possess adaptive immunity and rely solely on innate immunity. In this study, we examined whether ectopic overexpression of NSD, the *Drosophila* homolog of human NSD1, disturbs innate immunity, since deregulation of the immune system due to the loss-of-function of NSD1 has been reported in SOTOS patients and certain types of human tumor cells. In addition, we observed transcriptional perturbation of various immune-response-related genes by NSD overexpression (unpublished data). Recently, the insect immune response was reported to be epigenetically modulated [11], indicating a potential role of NSD in the innate immune system. Thus, because the fat body of *Drosophila* is a central organ for immune responses, similar to the liver of humans, we explored the role of an epigenetic writer of H3K36, NSD, in humoral immunity when overexpressed in the fly fat body.

NSD is an epigenetic modulator that catalyzes H3K36 mono- and di-methylation. Our results in Figure 2 demonstrate that the overexpression of NSD induces the expression of AMPs, more preferentially in the IMD pathway, in the normal condition without bacterial infection. Similarly, a recent study by Wu and Yan (2022) [27] reported that increased heterochromatin formation by HP1a in the fat body was associated with specific upregulation of IMD-mediated AMPs even before infection, providing insights into the epigenetic strategies of *Drosophila* innate immunity against Gram-negative bacterial infection. Given that NSD is enriched in heterochromatin and interacts with HP1a [28], it is plausible that NSD plays an essential role in the IMD pathway via HP1a-mediated heterochromatin formation. Our findings support this possibility and suggest that NSD-mediated H3K36 methylation may contribute to the upregulation of IMD pathway AMPs through the formation of HP1a-mediated heterochromatin. This provides a new perspective on the mechanisms underlying the regulation of *Drosophila* innate immunity and the role of NSD in this process. Further studies are needed to elucidate the specific molecular mechanisms involved in this process and to explore the potential therapeutic applications of targeting NSD in the context of infectious diseases.

The *Drosophila* humoral immune system responds to a microbial challenge by triggering the expression of AMP genes via NF-κB/Rel [29]. Two NF-κB signaling pathways control AMP gene expression: Dorsal/Dif activates the Toll pathway, and Relish activates the IMD pathway. Although each pathway responds to distinct microbial components and produces different sets of AMPs, it can induce the expression of overlapping subsets of AMP. Furthermore, the co-activation of different NF-κB factors displays synergistic activation of the representative target genes of the two pathways [30,31]. We chose three—*Drs*, *Dip*, and *AttA-AMPs*—and tested the effects of NSD overexpression on their transcription levels. Although all three peptide mRNAs were increased, the promoter of *AttA* was chosen for further analysis, as it displayed high activation by NSD and its expression was affected by both Toll and IMD signaling. NSD also induced a high level of transcriptional activation of *Drs*, a major target gene of the Toll pathway (Figure 2B). Thus, although we revealed that the *AttA* promoter is preferentially activated by NSD via the *Drosophila* NF-κB-like factor Relish, further investigation is needed to determine whether *Drs* activation is due to synergistic activation by IMD or the sole effect of Dorsal/Dif activation, which was not examined in this study.

The transcription factor NF-κB regulates a wide variety of genes, and most NF-κB-responsive promoters include multiple κB sites, which act synergistically to bring about dramatic increases in transcription levels [32]. Similar to other NF-κB-responsive promoters, the *AttA* promoter contains four putative κB sites [33]. However, we evaluated the effect of NSD on only two proximal NF-κB sites located at positions −118 and −46 to the transcription start site, because it has been reported that the proximal κB sites govern *AttA* induction by the Toll and IMD pathways, while the other two distal sites are less important. Thus, to identify the critical NF-κB site in the *AttA* promoter for NSD function, we used reporters driven by mutant *AttA* promoters containing deletions at two major proximal NF-κB sites (Figure 3A). Because other intact κB sites distally located at the *AttA* promoter could still respond to NSD, complete deletion of the NF-κB sites may further decrease the effect of NSD on promoter activity, or other activators may be involved in NSD-mediated transactivation.

However, the role of NSD1 in infectious diseases in humans has not been thoroughly studied. In contrast, NSD1 is best known as the causative gene for the congenital overgrowth disorder SOTOS [34] and is genetically or epigenetically deregulated (either inactivated or overexpressed) in other cancer types [35,36,37,38]. Recently, an association between NSD1 and NF-κB has been reported. A high level of NSD1 activates NF-κB, whereas reduced expression of NSD1 decreases NF-κB activation via the reversible lysine methylation of NF-κB [39]. NSD1 reportedly facilitates the epithelial-mesenchymal transition, migration, and invasion of paclitaxel-resistant breast cancer cells by regulating NF-κB [40]. In addition, children with SOTOS frequently suffer from infections, and NSD1 inactivation displays an immune-cold phenotype, indicating its potential association with immune disturbances. Thus, examining the effect of NSD overexpression on NF-κB activation in relation to the infection in the *Drosophila* model in this study seems relevant.

NF-κB transcription factors are central coordinators of immune and inflammatory responses and are evolutionarily well-conserved. Because of the conserved structure and function of NF-κB/Rel proteins across the animal kingdom, *Drosophila* has been extensively used to probe the molecular mechanisms of NF-κB activation and its role in inflammation, infection, and disease. Thus, understanding the modulation of immune response signaling due to NSD protein overexpression in the fly fat body, as observed in this study, is expected to contribute to the elucidation of the molecular mechanisms of NSD1 malfunctions in various NSD1-associated human diseases, such as SOTO1 and NSD1 duplication-related disorders.

## 4. Materials and Methods

### 4.1. Fly Species and Bacterial Strains

The *Drosophila melanogaster* flies used for the experiments were cultured in a standard medium at 25 °C and 60% relative humidity. The flies used for the experiments were as follows: *w1118*, *UAS-NSD* (BL22044), *Cg-Gal4*, and *Da-Gal4*, all from the Bloomington Stock Center (Bloomington, USA). The *Pe* strain was purchased from the Leibniz Institute DSMZ-German Collection of Microorganisms and Cell Cultures (Braunschweig, Germany) and cultured in Luria–Bertani (LB) medium.

### 4.2. RNA Preparation and qPCR

Fly RNA was extracted using TRIzol reagent (Invitrogen, Carlsbad, NM, USA), according to the manufacturer’s guidelines, and quantified using a Nanodrop ND-1000 spectrometer. After treatment with DNase I (Takara, Shiga, Japan) to digest genomic DNA, cDNA was synthesized using the HiSenScript RH(-) RT premix kit (iNtRON Biotechnology, Seongnam, Republic of Korea). Moreover, qPCR was performed using the SYBR Green Master Mix reagent (Applied Biosystems, Foster, CA, USA). PCR quantification was performed using the 2^-∆∆Ct^ method, and the numbers were normalized to the Rp49 transcript levels. The primer sequences used in this study are listed in Table 1. Each experiment was repeated at least thrice.

### 4.3. Immunohistochemistry

In preparation for *Drosophila* larval fat body immunofluorescence, larvae were dissected in phosphate-buffered saline (PBS) and fixed with 4% paraformaldehyde in PBS for 1 h at room temperature. After fixation, the tissues were washed thrice with PBS, permeabilized with 0.3% Triton X-100 in PBS (PBT), and blocked with 5% bovine serum albumin for 1 h each. After blocking, the tissues were incubated overnight with primary antibodies diluted in blocking solution (1:100) at 4 °C. After treatment with primary antibodies, the tissues were washed three times with 0.3% PBT and incubated with anti-mouse and anti-rabbit secondary antibodies conjugated to Alexa-488 and -555 (Invitrogen), which were diluted in blocking solution (1:200) at room temperature for 1 h. The cells were washed thrice with 0.3% PBT and incubated with 4′-6-diamidino-2-phenylindole for 5 min. Tissues were mounted with 90% glycerol and observed under an LSM laser-scanning confocal microscope (Carl Zeiss, LSM800, Oberkochen, Germany).

### 4.4. Plasmid Vector Cloning and Transient Transfection of S2 Cells

All luciferase reporter constructs were constructed using p*GL3*-Basic vector (Promega, Madison, WI, USA). To generate p*AttA*_*Luc*, the −488 to +30 region of the *AttA* promoter was amplified using PCR. To delete the *AttA* promoter sequence, we utilized the Phusion Site-Directed Mutagenesis Kit from Thermo Fisher (Waltham, MA, USA), following the manufacturer’s instructions. The mutagenic oligonucleotides listed in Table 1 were used to introduce site-directed deletions into the *AttA* reporter constructs.

As a preparation for transient transfection, one day before transfection, *Drosophila* S2 cells were seeded in a 12-well plate and incubated overnight at 28 °C. After transfection using the *pAttA* reporter plasmids and the NSD-expressing effector plasmid in a 1:5 ratio, copper sulfates were added for 24 h to induce the expression of the cotransfected *MT* promoter-driven NSD gene. All data were normalized to the internal control p*Ac5C*-*RL*. Transfection was performed using Lipofectamine 3000 reagent according to the manufacturer’s instructions (Thermo Fisher, Waltham, MA, USA).

### 4.5. Luciferase Assay

For the luciferase assay, S2 cells were transfected for 24 h and harvested. The washed cells with PBS were centrifuged at 3000× *g* at 4 °C for 5 min, and the supernatant was removed. Diluted passive lysis buffer was added to the cell pellet, which was then incubated at room temperature on a rocking platform for 30 min. After incubation, the cell samples were transferred to a 96-well plate, and luciferase activity was measured using the Reporter Assay System, according to the manufacturer’s instructions (Promega, Madison, WI, USA).

### 4.6. Western Blot

To prepare the western blot assay, wandering stage third instar larvae and S2 cells were homogenized in 1 × Laemmli sample buffer, and the lysates were incubated at 4 °C for 30 min. After incubation, samples were centrifuged at 10,000× *g* at 4 °C for 30 min. After centrifugation, the supernatants were harvested, and protein concentrations were measured using the Protein Assay Dye Reagent (Bio-Rad, #5000006, Hercules, CA, USA). After quantification, the protein samples were diluted in sodium dodecyl sulfate (SDS) gel loading buffer with DTT (100 mM) and heated at 95 °C for 10 min before separation by SDS-polyacrylamide gel electrophoresis. Proteins were transferred to polyvinylidene difluoride membranes (Millipore, Burlington, MA, USA) and blocked with 5% skim milk in Tris-buffered saline containing 0.01% Tween 20 (TBST). After blocking, the proteins were probed with anti-NSD (GenScript, Piscataway, NJ, USA), anti-actin (JLA-20, Developmental Studies Hybridoma Bank, Iowa City, IA, USA), anti-Relish C-term (anti-Relish-C 21F3, Developmental Studies Hybridoma Bank, Iowa City, IA, USA), anti-Relish N-term (Rabbit Anti-Relish, Ray Biotech, Peachtree Corners, GA, USA), and anti-H3K36Me2 (Abcam, Cambridge, UK) antibodies at 4 °C overnight in 5% bovine serum albumin (Sigma-Aldrich, St. Louis, MO, USA). After washing three times with TBST, the membranes were treated with horseradish peroxidase-conjugated secondary antibodies at room temperature for 2 h. Protein band signals were detected using an LAS-4000 luminescent image analyzer (Fujifilm, Tokyo, Japan).

### 4.7. Natural Pe Infection of Drosophila Larvae and its Effect on Larval Survival Rates

*Drosophila* larvae were infected as previously described, with modifications [24]. Approximately 100 third instar larvae were placed in a 2-mL tube containing 200 µL of concentrated bacterial pellet (OD_600_ 200) from an overnight culture and 400 µL of crushed banana. The larvae, bacteria, and banana were mixed in a microfuge tube; the tube was incubated at room temperature for 30 min, and the mixture was then transferred to a grape agar plate and incubated at 29 °C with 60% humidity and normal light–dark cycles. Larvae were collected at different time intervals after infection.

### 4.8. Statistical Analysis

All data were organized using Excel (Microsoft), and statistical analyses were performed using the Mann–Whitney U test for two-group comparison tests (SPSS 28 software, IBM). In the data, the bar graphs are expressed as means ± standard error. Any p-values less than 0.05 (** *p* < 0.05, *** *p* < 0.005) were considered to be statistically significant. Data obtained in the survival assay were summarized as a Kaplan–Meier graph and analyzed statistically using the log-rank approach.

## Figures and Tables

**Table 1:** Primer pairs for quantitative RT-PCR and cloning of site-directed deletions.

Name	Sequence (5′→3′)
NSD_F	TCCATCGTGTGGGCATATT
NSD_R	TGCATCATCCTTGAGTTTC
Drs_F	GTTCGCCCTCTTCGCTGTCC
Drs_R	CCACTGGAGCGTCCCTCCTC
Dip_F	GCTGCGCAATCGCTTCTACT
Dip_R	TGGTGGAGTGGGCTTCATG
Att_F	CACAATGTGGTGGGTCAGG
Att_R	GGCACCATGACCAGCATT
rp49_F	TACAGGCCCAAGATCGTGAA
rp49_R	GTTCGATCCGTAACCGATGT
ΔToll_F	GCTTTGATAAGGCATCCAGGCC
ΔToll_R	GCTCAGATGTGATGGTGGTTTACTTC
ΔImd_F	GCATCTTGAGGTATAAAACCGATGCATTG
ΔImd_R	CTGATGATTGACATGATTCATCTGATTGC

## Data Availability

The data presented in this study are available on request from the corresponding author.
